# Psychosocial Impacts of COVID-19 on Healthcare Workers During the Nationwide Partial Lockdown in Vietnam in April 2020

**DOI:** 10.3389/fpsyt.2021.562337

**Published:** 2021-07-20

**Authors:** Thao Thanh Nguyen, Xuan Thi Thanh Le, Nguyen Thao Thi Nguyen, Quang Nhat Nguyen, Huong Thi Le, Quan Thi Pham, Nhung Kim Thi Ta, Quynh Thi Nguyen, Anh Ngoc Nguyen, Men Thi Hoang, Hai Quang Pham, Linh Gia Vu, Anh Mai Luong, David Koh, Trang Ha Nguyen, Bach Xuan Tran, Carl A. Latkin, Cyrus S.H. Ho, Roger C.M. Ho

**Affiliations:** ^1^School for Preventive Medicine and Public Health, Hanoi Medical University, Hanoi, Vietnam; ^2^Duke School of Medicine, Duke University, Durham, NC, United States; ^3^Institute for Global Health Innovations, Duy Tan University, Da Nang, Vietnam; ^4^Nuffield Department of Medicine, University of Oxford, Oxford, United Kingdom; ^5^Faculty of Medicine, Duy Tan University, Da Nang, Vietnam; ^6^Center of Excellence in Evidence-based Medicine, Nguyen Tat Thanh University, Ho Chi Minh City, Vietnam; ^7^Vietnam Health Environment Management Agency, Ministry of Health, Hanoi, Vietnam; ^8^Pengiran Anak Puteri Rashidah Sa'adatul Bolkiah (PAPRSB), Institute of Health Science, University Brunei Darussalam, Gadong, Brunei; ^9^Saw Swee Hock School of Public Health (SSH) School of Public Health, National University of Singapore, Singapore, Singapore; ^10^Bloomberg School of Public Health, Johns Hopkins University, Baltimore, MD, United States; ^11^Department of Psychological Medicine, National University Hospital, Singapore, Singapore; ^12^Department of Psychological Medicine, Yong Loo Lin School of Medicine, National University of Singapore, Singapore, Singapore; ^13^Institute for Health Innovation and Technology (iHealthtech), National University of Singapore, Singapore, Singapore

**Keywords:** COVID-19, mental health, psychosocial impact, healthcare worker, Vietnam

## Abstract

**Background:** The psychosocial impact of COVID-19 is greater among healthcare workers (HCWs) than the general population. This study aims to identify psychosocial problems faced by HCWs in Vietnam during the national partial lockdown between 1 and 22 April 2020 and to identify risk factors associated with psychosocial issues among this population.

**Methods:** A cross-sectional study was conducted in the second week of April 2020 during the national lockdown in Vietnam. Snowball sampling technique was used to recruit participants through web-based surveys. The Impact of Events Scale-Revised (IES-R) was used to assess the impact of COVID-19 on HCWs through online surveys.

**Results:** Of the 349 HCWs, we found 22.6% reported psychosocial problems. Most of participants reported having exposure to COVID-19 daily (48.7%). The majority of them also felt that their job put them at risk of SARS-CoV-2 infections (90.3%) and expressed fear of potential infection (85.7%). Despite COVID-19 risks, 95.4% of participants, however, expressed their willingness to continue working at their current health facility. In addition, 94.8% of participants believed if they or their family members had been infected, their agency leaders would have provided them with appropriate medical care. Lastly, HCWs who worked in the internal medicine department who did not take care of COVID-19 patients or expressed fear of becoming infected were more likely to have higher total IES-R scores.

**Conclusion:** Our findings suggest that the support of healthcare leaders and assurance of care might be helpful in mitigating the psychological effects of COVID-19 among HCWs in Vietnam. These resources should be tailored to HCWs who are working in different areas of health services, including staff who are not working directly with COVID-19 patients. In addition, psychosocial health resources should be provided for not only physicians but also nursing staff.

## Introduction

The present coronavirus disease 2019 (COVID-19) due to SARS-CoV-2 has resulted in widespread reports of worsening mental health (1–4). In particular, rising numbers of COVID-19 cases and the consequent institutionalized stay-at-home orders exacerbated feelings of isolation along with fear of potential infections, thereby contributing to the increasing issues around psychosocial well-being. A study in China found that panic disorders, anxiety and depression were the most widespread during this pandemic (1). Other common psychological impacts included anger, guilt, grief and loss, post-traumatic stress disorder (PTSD), and stigmatization (5, 6).

The institution of public health measures, such as stay-at-home orders, has played a crucial role in delaying the spread of infections and alleviating pressure on healthcare systems particularly in low-to-middle income countries (LMICs), such as Vietnam, where critical care resources are already limited. Due to Vietnam's rigorous public health interventions, as of the end of April 2020, there were only 270 confirmed COVID-19 cases with zero deaths nationally. Nevertheless, the stringent social distancing measures, combined with the stress of working in high-risk, resource-poor settings have put people, particularly healthcare workers (HCWs) at risk of developing psychosocial disorders (7). These issues have been further compounded by the absence of mental health resources for HCWs—in particular interventions to combat stress, burnout, and PTSD during early stages of the outbreak (8).

The higher rates of mental problems among HCWs during the COVID-19 pandemic have been widely documented (9, 10). Previous studies among HCWs treating COVID-19 patients in China found higher levels of anxiety, stress, and self-efficacy based on sleep quality and social support (11). Another study found that non-frontline nurses were more likely to suffer psychological consequences than frontline nurses (12), highlighting the varying adverse mental health effects on all HCWs regardless of their direct contact with COVID-19 patients. Furthermore, poor mental health among HCWs has been apparent in past outbreaks, with previous studies demonstrating poor psychosocial outcomes even 1 year after the SARS outbreak in 2003 (13) and MERS in 2005 (14). In the present pandemic, a greater understanding of risk factors to support the development of early intervention for mental health illnesses among HCWs will thus be crucial to ensure the sustainability of our healthcare system in the years to come.

In this study, we examined the psychosocial impact of COVID-19 on HCWs during the first national lockdown in the history of Vietnam in April 2020. We aim to identify rates of psychosocial disorders among HCWs in Vietnam, trends contributing to these rates, and opportunities to improve the psychosocial health of this population. A better understanding of mental health needs among HCWs will help further ensure the well-being of this critical workforce during the present COVID-19 and potentially future pandemics in Vietnam.

## Methods

### Study Setting and Participants

A cross-sectional study was conducted on the second week of April 2020 during the national lockdown in Vietnam. During this time, all Vietnamese people were highly encouraged to stay at home and physically distance to prevent COVID-19 outbreak. By the end of April 2020, there were 270 cases of COVID-19 in Vietnam. Of note, Vietnam's healthcare system provides services at commune, district, provincial and central levels, and in this study, participants were all HCWs associated with hospital facilities—not just physicians and nurses. They were recruited according to the following eligibility criteria: (1) agreement to participate in the study through online informed consent forms, (2) ability to access the web-based surveys, and (3) ability to read and respond to the questionnaire.

### Sample and Sampling

In this study, we used a snowball sampling technique to recruit participants; active participants were asked to recruit other subjects for the study. This sampling method was considered to be suitable to study small groups of specialized workers who are likely to already know each other (15). At the beginning of the recruitment process, a core group of Hanoi Medical University medical doctors were established to conduct recruitment. The selected group reflected the diversity of study subjects with regards to age, gender, and occupation throughout the country. By distributing the questionnaire link, the core group disseminated the survey to their close contacts and other groups through social media (e.g., Facebook or Zalo). Study participants were asked to invite their colleagues and other HCWs across the country to take the survey. Using this approach, we recruited a total of 349 HCWs including those in hospitals, healthcare centers and medical universities throughout all 63 provinces of Vietnam during 1 week of data collection.

### Instruments and Measurements

The introduction of the study and informed consent were presented on the first page of the survey. After agreeing to participate the study, the respondents answered questions on the following topics:

#### Demographic Characteristics

The demographic characteristics included region, level of hospital and department which HCWs are working, gender, marital status, people that respondents were living with, education level, occupation, age, and duration of career.

#### Risk of Exposure to COVID-19

Participants self-reported their risk of exposure to COVID-19, which included exposure level (every day, several times per week, seldom, or unknown). Participants also answered eight questions about their perception on risk of COVID-19 which rated by using a five-point Likert scale from one representing “Strongly disagree” to five representing “Strongly agree”.

#### Psychological Impacts

To evaluate the psychosocial impacts of COVID-19, we used the Impact of Event Scale-Revised (IES-R) which evaluates post-traumatic stress symptoms (PTSD) or acute stress of participants and its severity after exposure the traumatic event during the national lockdown. There are 22 questions which were rated from 0 (Not at all) to 4 (Extremely). The total score of IES-R scale (15) was calculated by adding the scores of each question; it ranged from 0 to 88—a cutoff score of 33 or greater was considered positive for PTSD. In addition to providing a total score, the IES-R scale also contained three sub-scales for (1) Intrusion (8 items), (2) Avoidance (8 items), and (3) Hyperarousal (6 items). The score of each subscale was calculated by taking the average of total items in this subscale, which ranged from 0 to 4 (16). The IES-R total scores were interpreted using the following breakdown: 0–23 was normal, 24–32 was considered to be clinically concerning, 33–36 was classified as PTSD, and 37+ was represented extreme symptoms. The IES-R scale has been validated to measure levels of PTSD in both Western and Asian populations (17, 18). In this study, the Cronbach's alpha was 0.94.

### Data Analysis

STATA 15.0 (StataCorp LP, College Station, TX) was used to analyze the data. Descriptive statistics were adopted to calculate frequency, percent, mean and standard deviation. Inferential statistics were applied to perform the comparison among three subject groups by the *t*-test or Mann-Whitney test for quantitative variables and by the Fisher-exact test or chi-square test for qualitative variables. Ordered logistic regression and multivariable regression models were applied to identify factors associated with the psychological impacts of participants during COVID-19 lockdown. The outcomes of regression models were the severity of PTSD and three subscales of IES-R scale (Intrusion, Avoidance, and Hyperarousal). Independent variables included demographic characteristics and risk of exposure to COVID-19. To obtain reduced models, stepwise forward selection strategies were utilized with a log-likelihood ratio test at a p-value of 0.2. Statistical significance was defined at a *p*-value of less than 0.05.

### Ethical Consideration

This study was approved by the Ethics Review Committee at the Institute for Preventive Medicine and Public Health, Hanoi Medical University, dated March 28, 2020. The purpose of research and informed consent forms were provided through the web-based platform. Participation was voluntary, and anonymity was assured. Participants were informed they could decline to participate or withdraw from the online survey at any time.

## Results

The socioeconomic characteristics of the participants (*n* = 349) are presented in Table 1. Thirty-nine percent of participants were male. The majority of participants worked in provincial and central hospitals (30.7 and 33.8%, respectively), were hysicians (57.0%) and were married (75.1%). There were 57.9% of participants had attained a University level of education or lower. The mean age was 35.2 (SD = 8.8) years. The mean career duration was 10.3 (SD = 8.2) years.

**Table 1:** Socioeconomics characteristics of participants.

	**Posttraumatic stress disorder (IES-R)**				**Total**		***p*-value**
	**No**		**Yes**				
	**n**	**%**	**n**	**%**	**n**	**%**	
**Total**	306	87.7	43	12.3	349	100.0	
**Region**							
Northern	224	73.2	33	76.7	257	73.6	0.80
Central	63	20.6	7	16.3	70	20.1	
South	19	6.2	3	7.0	22	6.3	
**Level of hospital**							
Central level	106	34.6	12	27.9	118	33.8	0.48
Provincial level	93	30.4	14	32.6	107	30.7	
District health center	34	11.1	8	18.6	42	12.0	
Others	73	23.9	9	20.9	82	23.5	
**Gender**							
Male	114	37.3	22	51.2	136	39.0	0.08
Female	192	62.7	21	48.8	213	61.0	
**Marital status**							
Single / Separated/Widowed	77	25.2	10	23.3	87	24.9	0.79
Married	229	74.8	33	76.7	262	75.1	
**Living with**							
Family/friends	279	91.2	37	86.0	316	90.5	0.27
Alone	27	8.8	6	14.0	33	9.5	
**Education**							
University and lower	171	55.9	31	72.1	202	57.9	0.04
Higher than university	135	44.1	12	27.9	147	42.1	
**Occupation**							
Doctor	180	58.8	19	44.2	199	57.0	0.19
Nurse	69	22.5	13	30.2	82	23.5	
Others	57	18.6	11	25.6	68	19.5	
**Department**							
Emergency-Intensive care	23	7.5	5	11.6	28	8.0	0.85
Internal medicine	38	12.4	5	11.6	43	12.3	
Surgery-Obstetrics-Pediatrics	37	12.1	8	18.6	45	12.9	
Imaging Diagnosis-Scientific laboratory - Clinic	44	14.4	4	9.3	48	13.8	
Administrative offices	47	15.4	7	16.3	54	15.5	
Infectious disease-Infection control	14	4.6	2	4.7	16	4.6	
Preventive medicine-Public health-Nutrition	41	13.4	4	9.3	45	12.9	
Others	62	20.3	8	18.6	70	20.1	
	**Mean**	**SD**	**Mean**	**SD**	**Mean**	**SD**	***p*****-value**
**Age** (unit: years)	35.0	8.5	36.3	10.7	35.2	8.8	0.54
**Duration of career** (unit: years)	10.1	7.9	11.7	9.7	10.3	8.2	0.44

Table 2 shows the self-reported and perceived risk of exposure to COVID-19. There were no significant difference in HCWs' risk of exposure to COVID-19 and the presence of PTSD. Over half of participants (48.7%) reported risk of COVID-19 exposure every day. On the contrary, 13.5% of HCWs reported they were not at risk of COVID-19 exposure, and 13.8% reported that they were unaware of their risk. Most of HCWs agreed to continue working at their current health facility, despite a possible risk of COVID-19 exposure (95.4%). Nearly all participants believed their agency leaders would provide them with medical services if they were infected with the virus (94.8%). Nevertheless, the majority of HCWs felt their jobs put them at risk of SARS-CoV-2 infections (90.3%) and reported fear of being exposed to COVID-19 (85.7%). The majority of participants reported their families perceived them to be at high risk for COVID-19 (82.8%).

**Table 2:** Risk of exposure to COVID-19.

	**Posttraumatic stress disorder (IES-R)**				**Total**		***p*-value**
	**No**		**Yes**				
	**n**	**%**	**n**	**%**	**n**	**%**	
**Risk of exposure to COVID 19**							
Do not expose to risk factors	38	12.4	9	20.9	47	13.5	0.53
Everyday	153	50.0	17	39.5	170	48.7	
Several times per week	32	10.5	4	9.3	36	10.3	
Seldom	42	13.7	6	14.0	48	13.8	
Do not know	41	13.4	7	16.3	48	13.8	
**Perception on risk of COVID 19**							
Accept to continue working at a current health facility, even though it may be contaminated with COVID 19	293	95.8	40	93.0	333	95.4	0.43
The agency leader will provide me with the necessary medical services if I am infected with COVID 19	291	95.1	40	93.0	331	94.8	0.47
Feel the job put you at high risk of being exposed to COVID 19	277	90.5	38	88.4	315	90.3	0.59
Fear of being infected COVID 19	261	85.3	38	88.4	299	85.7	0.59
My family believes that I am at high risk for COVID 19	255	83.3	34	79.1	289	82.8	0.49
Accept colleagues to quit their jobs because they are afraid of COVID-19 infection	138	45.1	20	46.5	158	45.3	0.86
Do not take care of COVID 19 patients	54	17.6	15	34.9	69	19.8	0.01
If infected with COVID 19, I believe that my chances of survival are low	40	13.1	12	27.9	52	14.9	0.01

**Figure 1:**
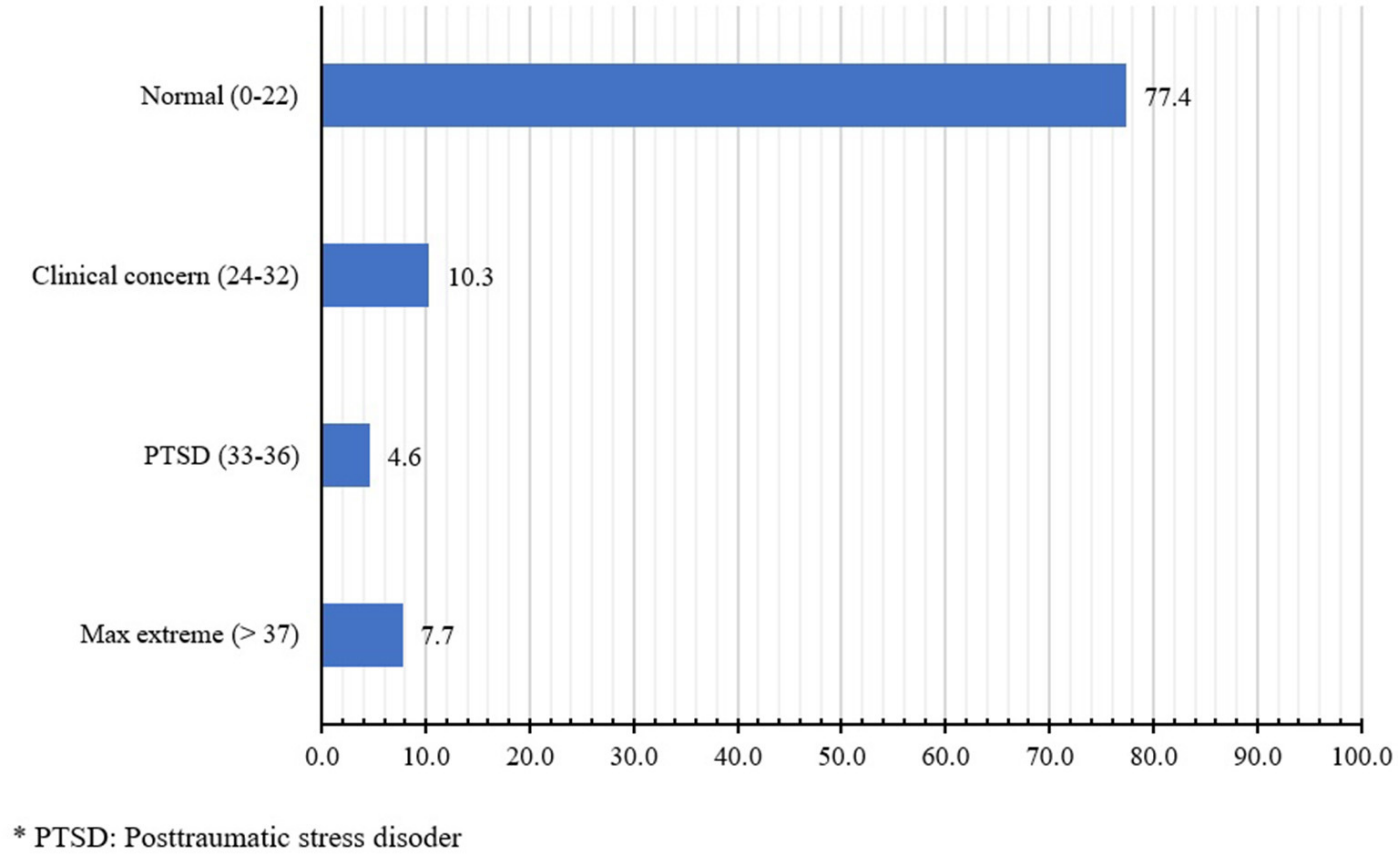
Levels of posttraumatic stress disorder impact by COVID-19 among HCWs.

Figure 1 presents levels of PTSD as indicated by participants' IES-R score. The psychosocial impacts of COVID-19 on HCWs were categorized into four groups: normal (77.4%), clinically concerning (10.3%), PTSD (4.6%) and extreme symptoms (7.7%). Table 3 shows the relationship between IES-R scores, the perceived risk of SARS-CoV-2 infections, and demographic characteristics of the participants. We found that Internal Medicine department staff were more likely to experience greater psychosocial effects of COVID-19 across the three IES-R subscales in comparison to their those in Emergency-Intensive Care. We also found participants who were not responsible for caring for COVID-19 patients and those who were fearful of SARS-CoV-2 infections were more likely to have higher total IES-R scores across all three domains. Regarding the education levels, we found that those who had attained a degree greater than University were more likely to be affected by the psychosocial effects of COVID-19; however, they were less likely to suffer from intrusion, avoidance and hyperarousal symptoms.

**Table 3:** Associated factors of psychological and social impacts of COVID-19.

	**IES-R level**		**IES-R scale**					
			**Intrusion subscale**		**Avoidance subscale**		**Hyperarousal subscale**	
	**OR**	**95% CI**	**Coef**.	**95% CI**	**Coef**.	**95% CI**	**Coef**.	**95% CI**
**Age**			0.01^**^	0.00; 0.02				
**Education** (vs. University and lower)								
> University	0.56^*^	0.32; 1.00	−0.24^***^	−0.41; −0.07	−0.14^*^	−0.30; 0.02	−0.21^***^	−0.36; −0.06
**Gender (vs Male)**								
Female							−0.14^**^	−0.27; −0.00
Marital status (vs. Single/Separated/Widowed)								
Marriage			0.17^*^	−0.02; 0.36				
Department (vs. Emergency-Intensive care)								
Internal medicine	2.02^**^	1.06; 3.85	0.24^**^	0.03; 0.45	0.30^***^	0.12; 0.48	0.35^***^	0.16; 0.54
Surgery-Obstetrics-Pediatrics							0.14	−0.05; 0.34
Administrative offices			0.14	−0.06; 0.34				
Level of hospital (vs. Central level)								
Others					−0.13^*^	−0.28; 0.02		
**Occupation (vs Doctor)**								
Nurse					0.14^*^	−0.03; 0.32		
Others					0.12	−0.05; 0.29		
**Years of career** (years)	1.04^**^	1.01; 1.08			0.01^***^	0.00; 0.02	0.01^**^	0.00; 0.02
**Perception on risk of COVID-19**(Agree vs Not Agree)								
Feel the job put you at high risk of being exposed to COVID-19					−0.14	−0.36; 0.07		
Fear of being infected COVID-19	2.23^*^	0.90; 5.53	0.28^**^	0.06; 0.49			0.21^**^	0.02; 0.40
Do not take care of COVID-19 patients	1.71^*^	0.93; 3.16	0.19^**^	0.00; 0.38	0.27^***^	0.11; 0.43	0.25^***^	0.08; 0.42
Accepting of colleagues who quit their jobs due to fear of SARS-CoV-2 infection					0.12^*^	−0.01; 0.25		
My family believes that I am at high risk for COVID-19			0.14	−0.06; 0.34				

*** *p < 0.01;*

** *p < 0.05;*

* *p < 0.1*.

## Discussion

The association between emerging infectious diseases and mental illness has been demonstrated during outbreaks in the past and is observed in this study (19, 20). Psychosocial interventions should prioritize HCWs, who have been found to experience worse mental health during these crises than the general population (9). In this study, we assessed the perceived risks of COVID-19, its psychosocial impact on HCWs during the partial lockdown in Vietnam in April 2020. We found that 22.6% of Vietnamese HCWs had reported psychosocial problems (Figure 1). Approximately half (48.7%) of participants reported exposure to COVID-19 daily, and the majority felt that their job put them at risk of SARS-CoV-2 infections (90.3%) and expressed fear of infection (85.7%). Nevertheless, we found that nearly all participants (95.4%) were willing to continue working at their current health facility despite the possible exposure to COVID-19. A similarly high number of participants (94.8%) believed that if they became infected, their agency leaders would provide them with appropriate medical care. Lastly, we found that those in the internal medicine department, those who reported not having to take care of COVID-19 patients, and those who expressed fear of becoming infected with the virus were more likely to have higher total IES-R scores.

The overall percentage of HCWs who reported having psychosocial problems in this study (22.6%) is lower than the rates found in heavily endemic countries, such as China (37%), but is higher than other Asian countries, such as Singapore (<20%) and India (<10%), during early stages of the COVID-19 pandemic (21). Nevertheless, the majority of participants expressed fear of SARS-CoV-2 infection (83.7%), felt that their job put them at risk of COVID-19 exposure (89.7%), and reported that their families believed they were at a high risk for COVID-19 exposure (80.9%). Half of the participants (51.4%) reported daily exposure to COVID-19. Given these high rates of reported COVID-19 exposure and fear of infections, the relatively low prevalence of psychosocial disorders is a source of optimism.

It is worth noting we did identify a positive relationship between fear of SARS-CoV-2 infection and IES-R score, an expected association given the well-established relationship between fear and PTSD (22). Nevertheless, regarding the reported exposure to COVID-19, we found that HCWs who did not have to take care of COVID-19 patients were more likely to have higher IES-R scores. This result was reported in a previous study in China (12). In our study, we also found that differences in educational level, job title and family condition between those selected to work with COVID-19 patients might contributed to the observed perception of risks of exposure and of the consequent psychological impact. Other possible explanations may include the desensitization among those on the “frontlines” associated with their routine interactions with COVID-19 patients, which could reduce their anxiety levels. Lastly, these findings may be a result of differing effects of concrete vs. theoretical risks in which the uncertainty of COVID-19 exposure could produce greater levels of anxiety than the accepted known threats of exposure.

In spite of high levels of fear, agency support appears to be an important factor for the relatively low psychosocial impact on HCWs. Nearly all participants (95.4%) were still willing to continue working at their current health facilities despite the possible risk of COVID-19 exposure, and 94.8% believed their agency leader would provide necessary medical services should they become infected. While the economic instability may play a role in these responses, these data highlight the potential impact of providing proper overall support for HCWs in the management of psychosocial disorders among this population. It might be worth considering extending such support and healthcare coverage to family members of HCWs because transmission to family members has been found to be a significant source of anxiety for HCWs (23). Given that 82.8% of participants reporting that their family believed they have high risk of COVID-19 exposure, these measures would provide further reassurance to family members and could relieve the pressure from the social stigma associated with HCWs' profession (24).

The relatively low psychosocial impact of COVID-19 on HCWs was also likely mediated by low rates of infections in Vietnam. With only 270 confirmed COVID-19 cases by the end of April 2020, Vietnam is among the global leaders with regards to its pandemic preventative strategies. Between March and April 2020, Bach Mai Hospital, the country's national hospital in Hanoi, had 19 positive cases of COVID-19 (25). Since the virus's initial introduction into Vietnam, there have only been four reported COVID-19 cases in HCWs as of April 2020 (25). This is much lower than in the U.S. with over 10,000 COVID-19 cases among HCWs (26, 27) and in China where 3,300 HCWs have been infected as of early March (23). Previous studies have found that the availability of personal protective equipment (PPE) and increasing work demands is among the primary concerns of HCWs regarding COVID-19 exposure (28). Given the low number of cases in Vietnam and the country's early preparation for the pandemic (29), these pressures were adequately mitigated, potentially contributing to our observed lower rates of psychosocial disorders among HCWs.

We also found Internal Medicine staff had significantly higher IES-R scores than their colleagues in the Emergency-Intensive Care department. These differences were likely mediated by greater preparation in anticipation of COVID-19-related needs by the Emergency-Intensive Care department. While preparedness in the intensive care department is critical, only a portion of hospitalized patients would require such a level of care (30). In fact, increasing pressure related to COVID-19 is distributed throughout the hospital system. In our study, we found that even those who did not take care of COVID-19 patients were psychosocially affected. Therefore, although greater effort to ensuring appropriate support for departments who might demonstrate higher needs of psychological support, such as the Internal Medicine reported in our study, healthcare systems should be prepared to provide adequate support to all HCWs regardless of the level of care and direct interactions with COVID-19 patients.

Moreover, we found that nurses had significantly higher IES-R scores on the avoidance subscale than physicians, which echoes findings from a similar study among HCWs during the H7N9 influenza outbreak in China in 2015–16 (31). These results are likely related to the higher degree in which nurses are on the “frontlines” than physicians due to their greater levels of patient interactions on a daily basis. In addition to disease prevention measures, greater provision of mental health screenings and other psychological or safe social services and activities could better address psychological needs for nurses during this high-stress time.

Several limitations of this study should be noted. First, the snowball sampling technique used in this study did not permit calculation of sampling error; caution should be taken in generalizing these findings to other settings. Second, the cross-sectional study design did not allow the identification of cause-effect relationships. Third, despite our effort in sampling to ensure the diversity of participants, our respondents were not randomly selected. Further, while IES-R questions have been validated in the evaluation of PTSD among both Western and Asian populations, participants' reports of risk of exposure and risk perception were self-reported. Therefore, even though the surveys were anonymized, social-desirability and recall bias might have impacted participants' responses.

## Conclusions

In outbreak settings, HCWs experience the brunt of the psychosocial effects. In this study, however, we found a relatively small number of HCWs self-reporting psychosocial problems associated with risk of COVID-19 exposures during the Vietnam's national lockdown in April 2020. This low rate could be attributed to a combination of factors, including the national pandemic response strategies, greater institutional support and lower rates of infections. Nevertheless, greater effort is needed to ensure proper access and adequate provision of psychological services for HCWs, especially nurses, HCWs in less acute settings, such as Internal Medicine staff, and those who might not have direct responsibility and interactions with COVID-19 patients. Further studies determining the effectiveness of specific forms of psychosocial support reflecting unique HCWs' needs and preferences are warranted.

## Data Availability Statement

The raw data supporting the conclusions of this article will be made available by the authors, without undue reservation.

## Ethics Statement

The studies involving human participants were reviewed and approved by the Review Committee of Hanoi Medical University dated 28 March 2020. Written informed consent for participation was not required for this study in accordance with the national legislation and the institutional requirements.

## Author Contributions

All authors contributed to conceptualizing the manuscript: conceptualization, QP, NT, QTN, and TrN: data curation, LV and HP: data analysis, ThN, XL, HL, AN, DK, and BT: methodology, HL, XL, BT, AL, and RH: supervision, ThN, XL, NN, and QNN: writing—original draft, ThN, XL, NN, QNN, HL, QP, NT, QTN, AN, MH, HP, LV, AL, DK, TrN, BT, CL, CH, and RH: writing—review and editing.

## Conflict of Interest

The authors declare that the research was conducted in the absence of any commercial or financial relationships that could be construed as a potential conflict of interest.
